# End-to-End Network for Pedestrian Detection, Tracking and Re-Identification in Real-Time Surveillance System

**DOI:** 10.3390/s22228693

**Published:** 2022-11-10

**Authors:** Mingwei Lei, Yongchao Song, Jindong Zhao, Xuan Wang, Jun Lyu, Jindong Xu, Weiqing Yan

**Affiliations:** 1 School of Computer and Control Engineering, Yantai University, Yantai 264005, China; 2 Computer School, Beijing Information Science and Technology University, Beijing 100101, China

**Keywords:** pedestrian detection, pedestrian tracking, re-identification, real-time

## Abstract

Surveillance video has been widely used in business, security, search, and other fields. Identifying and locating specific pedestrians in surveillance video has an important application value in criminal investigation, search and rescue, etc. However, the requirements for real-time capturing and accuracy are high for these applications. It is essential to build a complete and smooth system to combine pedestrian detection, tracking and re-identification to achieve the goal of maximizing efficiency by balancing real-time capture and accuracy. This paper combined the detector and Re-ID models into a single end-to-end network by introducing a new track branch to YOLOv5 architecture for tracking. For pedestrian detection, we employed the weighted bi-directional feature pyramid network (BiFPN) to enhance the network structure based on the YOLOv5-Lite, which is able to further improve the ability of feature extraction. For tracking, based on Deepsort, this paper enhanced the tracker, which uses the Noise Scale Adaptive (NSA) Kalman filter to track, and adds adaptive noise to strengthen the anti-interference of the tracking model. In addition, the matching strategy is further updated. For pedestrian re-identification, the network structure of Fastreid was modified, which can increase the feature extraction speed of the improved algorithm by leaps and bounds. Using the proposed unified network, the parameters of the entire model can be trained in an end-to-end method with the multi-loss function, which has been demonstrated to be quite valuable in some other recent works. Experimental results demonstrate that pedestrians detection can obtain a 97% mean Average Precision (mAP) and that it can track the pedestrians well with a 98.3% MOTA and a 99.8% MOTP on the MOT16 dataset; furthermore, high pedestrian re-identification performance can be achieved on the VERI-Wild dataset with a 77.3% mAP. The overall framework proposed in this paper has remarkable performance in terms of the precise localization and real-time detection of specific pedestrians across time, regions, and cameras.

## 1. Introduction

Video surveillance systems play a pivotal role in traffic, security, commercial, criminal investigation, and other fields [1,2,3]. With the rapid growth of the video data volume, how to quickly locate relevant data in unrelated mass data is a crucial issue. Manually searching or browsing for specific pedestrians in such a large amount of data is usually not feasible, as it is very time consuming, laborious, and most often unsuccessful. This makes the video surveillance system much less effective. Although the development of face recognition technology is more mature, in the case of the high-density crowds, low-resolution images, or lower camera angles and other circumstances, the use of face recognition technology often does not have the ideal effect. However, pedestrian re-identification technology can effectively locate and identify specific pedestrians in real-time surveillance videos, which is of great importance in the investigation of criminal cases and the search for missing persons. The core algorithms include object detection, multi-object tracking, and pedestrian re-identification.

In recent years, within the domain of visual analytics, there has been a surge in interest in visual surveillance searches, also known as forensic visual searches. However, there are few systems that perform search tasks in the context of surveillance. A related case is the IBM Smart Surveillance System [4], which is capable of indexing videos based on multiple search criteria, thus enabling various query types such as the primary object color, object size and type, and visual features of faces.

The visual search task is accomplished by detecting pedestrians in the video, generating a discriminative representation of specific pedestrians, and tracking them across time and regions. These representations are utilized in a query-by-example manner to compare the search images with the pool of detected pedestrians, so that a list of potential matches, sorted by similarity, is generated. In this paper, we implement an end-to-end pedestrian tracking and re-identification algorithm based on deep learning networks. It combines the YOLOv5-Lite detection algorithm and Deepsort tracking algorithm to detect specific pedestrians across time, regions, and cameras. It can achieve better results through a series of improvements, which can be commonly applied to intelligent monitoring and security systems.

The contributions of this paper are as follows:

- The network structure of the YOLOv5-Lite model is improved, and BiFPN is used for cross-scale feature fusion, which significantly improves the performance of pedestrian detection.
- The network structure of the Fastreid algorithm is improved to increase the speed of the extraction of pedestrian features; thus, the overall pedestrian re-identification efficiency is improved.
- The tracking strategy is optimized by using an improved Kalman filter algorithm and adding linear compensation. The improved Deepsort algorithm is used to track the re-identified pedestrian, and the tracking performance is significantly improved in all metrics.

Following is a brief summary of the rest of the paper. Section 2 introduces related works about object detection, pedestrian tracking and pedestrian re-identification. Section 3 describes the main framework of this paper. Section 4 details the improvements of the methods in detail. Section 5 presents the experimental results. Finally, Section 6 presents the conclusion.

## 2. Related Work

### 2.1. Object Detection

The development history of target detection can be categorised in AlexNet [5], with traditional feature descriptor algorithms dominating before 2012 and deep learning algorithms dominating after 2013. Traditional object detection algorithms search the entire image for regions with a class-specific maximum response using image descriptors such as HOG [6] and SIFT [7]. Numerous works based on CNN have been proposed as a result of the success of deep learning on object detection [8,9,10,11,12,13,14,15,16]. At present, CNN-based object detection can be classified into anchor-based and anchor-free methods.

#### 2.1.1. Anchor-Based Methods

Anchor-based methods employ the concepts of traditional sliding windows and proposal-based detectors, such as Fast R-CNN [17]. Their main approach is to a priori introduce anchor boxes, which are essentially pre-defined ideas, for bounding box regression. Anchor-based object detection can be broadly classified into *two-stage* and *one-stage* approaches. For two-stage detectors, anchors serve as regression references and classification candidates. Two-stage detectors have taken the lead thanks to the development of Faster R-CNN. It uses a region proposal network (RPN) and a region-wise prediction network (R-CNN) [18] to detect objects. Several algorithms are then put forward to improve its performance, including network structure redesign and improvement [19,20,21,22], the context and attention mechanism [23,24,25], pre-training strategies [26], feature fusion and data augmentation [27,28], loss function [29,30], and an improved proposal [31,32]. At present, the two-stage methods based on standard detection benchmarks still hold the most advanced results. For one-stage detectors, anchors act as the reference boxes for final selections. One-stage detectors have drawn significant interest due to their great computational efficiency since the emergence of SSD [33]. SSD distributes anchor boxes across multi-scale layers within the convolutional neural network to predict object categories and anchor box offsets directly. Since then, a large amount of work has been proposed to improve its performance in various areas, including merging context data from various layers [34], network architecture redesign [13,14], designing new loss functions [35], reintegration and the matching of anchor boxes [36], and feature enhancement and alignment [37,38]. Currently, one-stage methods can perform almost as well as two-stage methods while executing at a faster inference speed.

#### 2.1.2. Anchor-Free Methods

Unlike anchor-based detectors, anchor-free detectors do not require predetermined anchor boxes. In fact, there is a lengthy history of the anchor-free methods. There are primarily two types of anchor-free detectors suggested: *center-based methods* and *keypoint-based methods*. The former determines positives by using the object’s center points instead of anchor boxes, and then predicts the object bounding box for detection. The latter first detects a few pre-defined or self-taught keypoints and then generates bounding boxes to detect the objects. Early center-based anchor-free methods include YOLOv1 [39], DenseBox [40], and UnitBox [41]. Due to the relatively small number of positive samples, the recall rate for these detectors is low. In FCOS [9], all points within the object’s bounding box are considered positive samples. Besides detecting positive points, it also measures the distance between the points and the boundary box. For the purpose of detecting pedestrians with a fixed aspect ratio, CSP [42] only defines the object’s center points as positives. FoveaBox [43] treats the position in the center of the object as a positive with four distances to detect the object. It is simpler to predict the final class probability directly without the need for a centered voting. Keypoint-based anchor-free methods regard object detection as a keypoints localization problem, such as CornerNet [44], CenterNet [45], ExtremeNet [46] and RepPoint [47]. CornerNet recognizes the top-left and bottom-right corners of an object as a pair of critical points that define its bounding box. As opposed to a pair of keypoints, CenterNet expands CornetNet to a triplet to increase precision and recall. ExtremeNet generates the object bounding box by detecting four extreme points and one center point. RepPoints generates sets of points to represent objects using deformable convolutional networks (DCN) [48]. Additionally, some publications [49,50] attempt to extend the anchor-free idea to instance segmentation.

### 2.2. Multi-Object Tracking

Multi-Object Tracking (MOT) methods can be divided into tracking-by-detection methods and joint-detection-tracking methods. Tracking-by-detection methods [51,52,53,54,55] obtain the localization of objects before associating them with details about their appearance, motion, etc. With the rapid development of object detection techniques [10,14,44,45], tracking-by-detection methods have been a major task for MOT for many years. Simple Online and Real-time Tracking (SORT) [56] is a simple and effective method for tracking multiple objects using the Kalman filter and Hungarian algorithms. However, with more occlusion and various camera angles, the SORT algorithm’s effectiveness declines. To enhance the SORT algorithm, the developers of the Deepsort algorithm [57] added a new distance metric based on the “appearance” of the item. In recent years, a number methods of joint-detection-tracking [58,59,60,61,62] have been proposed to enhance detection and several other components, such as motion, embedding, and association models, jointly. The joint trackers provide equivalent performance at a minimal cost of computation. However, competition between various components and a lack of training data are the two main issues joint trackers are confronted with. The two issues place a cap on the maximum tracking precision. Therefore, the tracking-by-detection paradigm is still the best option in terms of tracking accuracy, as evidenced by the success of the most recent SORT-like frameworks [51,52,57,63].

### 2.3. Pedestrian Re-Identification

Pedestrian re-identification (Re-ID) is a technology used in video surveillance for public security and safety that seeks to recognize the same person in a collection of videos from non-overlapping camera perspectives. The process can be broken down into the following two steps: determining the embedding of features and matching them using a distance metric. It was discovered that merging image-level data with information on human parts could strengthen the robustness of Re-ID models [64,65,66]. Furthermore, numerous part-based strategies have shown significant advancements [3,67,68,69]. In order to handle partial occlusion, attention-based methods are becoming increasingly popular [70,71]. They construct useful video representations by choosing discriminative frames from video sequences. Several closely related works are as follows: Zhang et al. [70] proposed a deep graph model called Heterogeneous Local Graph Attention Networks (HLGAT) to model the inter-local and intra-local relationships with the attention mechanism in the completed local graph at the same time. Chen et al. [67] proposed a bidirectional interaction network for pedestrian Re-ID that considers multiple convolutional features as responses to various body part properties and exploits discriminative representations for person identities using inter-layer interaction. In [71], Second-Order Non-Local Attention Networks (SONA-Net) are proposed to directly model long-range relationships via second-order feature statistics for pedestrian re-identification. To address occlusion issues, Wu et al. [68] proposed a multi-level Context-aware Part Attention (CPA) model to learn discriminative and robust local part features for pedestrian Re-ID.

## 3. Overview of the Framework

**Figure 1:**
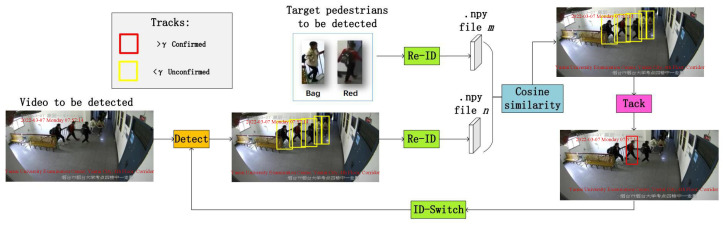
Overall system flow chart.

The overall process of the system consisting of YOLOv5-Lite, Deepsort and Fastreid algorithms is shown in Figure 1. Firstly, the target pedestrian image is intercepted; then, after the Fastreid feature extraction model, feature extraction is performed on the intercepted pedestrian base library to generate the corresponding .npy file, which is read into the video to be detected. YOLOv5-Lite object detection is used if the similarity is greater than the threshold γ, the target pedestrian is determined to be a target pedestrian, and then the target pedestrian is continuously tracked by the Deepsort algorithm, while the similarity is less than the threshold if the ID-Switch phenomenon (i.e., tracking failure) occurs while the Deepsort algorithm is tracking the target pedestrian; then, the YOLOv5-Lite algorithm is used again to detect all the pedestrians in the video and continue the next process. After this, the whole process is displayed through simple visualization.

## 4. Methods

### 4.1. Pedestrian Detection

Although YOLOv5-Lite is an improvement on YOLOv5, and the detection speed is significantly improved, the detection speed is not satisfactory in real-time video inference. The accuracy of the model trained on the training set still needs to be improved, and there may be problems of inconsistent feature information at each scale. Therefore, the model network structure still has room for improvement.

**Figure 2:**
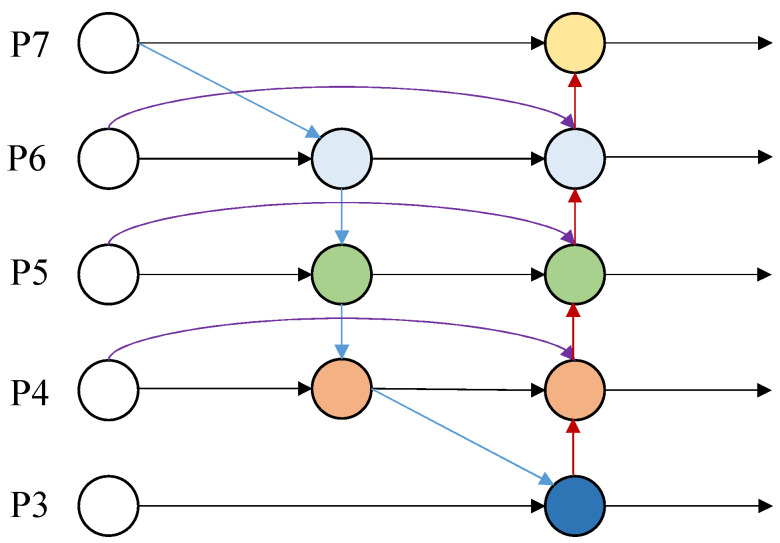
BiFPN [10] Network Architecture.

In order to accelerate the speed of real-time video inference, solve the possible inconsistency of multi-scale feature information in the model, and improve the accuracy of the model, BiFPN [10] (a weighted bi-directional feature pyramid network) is introduced, for with the structure is shown in Figure 2, which can make multi-scale feature fusion faster and more convenient. The main implementations are as follows.

- Adding residual connections: The intention is to enhance the representation of features by implementing simple residual operations, adding a jump connection between an input node and an output node at the same level, and fusing more features without considerably increasing the computational cost.
- Removing the nodes of single input edges: Since the nodes of single input edges are not fused, they have less information and do not contribute to a considerable extent to the final fusion, and removing them also reduces the computation.
- Weight fusion: In simple terms, this is the addition of a weight for each scale feature of the fusion, by adjusting the contribution of each scale to improve the detection speed by Fast-softmax [72]. The actual process is the fusion of the attention mechanism with FPN [73].

**Figure 3:**
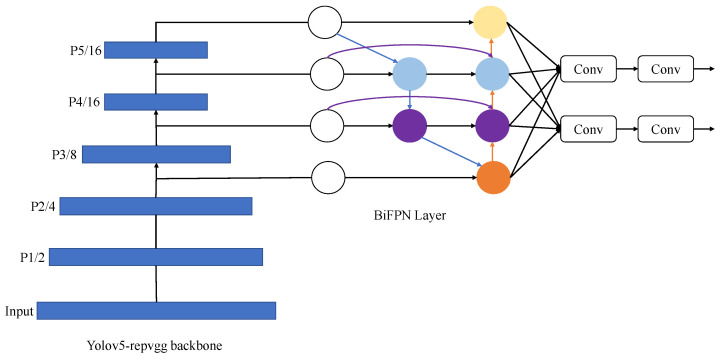
YOLOv5-Lite modified network structure diagram.

Overall, BiFPN achieves a combination of cross-scale bidirectional connectivity and fast normalization. For example, the two fusion features in Figure 3 at layer 6 are Equations (1) and (2).

(1) $$P_{6}^{td}=\mathrm{Conv}\left(\frac{w_{1}\cdot P_{6}^{in}+w_{2}\cdot \mathrm{Resize}\left(P_{7}^{in}\right)}{w_{1}+w_{2}+\epsilon }\right),$$

(2) $$P_{6}^{\mathrm{out}\;}=\mathrm{Conv}\left(\frac{w_{1}^{\prime }\cdot P_{6}^{\mathrm{in}\;}+w_{2}^{\prime }\cdot P_{6}^{td}+w_{3}^{\prime }\cdot \mathrm{Resize}\left(P_{5}^{\mathrm{out}\;}\right)}{w_{1}^{\prime }+w_{2}^{\prime }+w_{3}^{\prime }+\epsilon }\right),$$

where $P_{6}^{\mathrm{td}\;}$ is the intermediate characteristic of layer 6 in the top-down path, $P_{6}^{\mathrm{out}\;}$ is the output characteristic of layer 6 in the bottom-up path, and all other nodes are constructed in a similar manner. The Resize is usually a downsampling or upsampling operation; *w* is the parameter we learn to distinguish the importance of different features in the feature fusion process; and Conv is a convolutional op for feature processing.

One of the major features of BiFPN is to add weights to features at different scales. Compared with the traditional practice of directly stacking features at different scales, BiFPN can input different feature weights and let the network learn by itself, using Softmax-based fusion, as shown in Equation (3), $w_{i}$ and $w_{j}$ are learnable weights, and the softmax function is applied to normalize the weights to between 0 and 1.

(3) $$O=\sum_{i}\frac{e^{w_{i}}}{\sum_{j}e^{w_{j}}}.$$

For the branch model v5Lite-g of YOLOv5-Lite, the network header is modified by replacing all its Concat with BiFPN_Concat, and the model is changed, as shown in Figure 3.

### 4.2. Pedestrian Tracking

The Deepsort algorithm can be summarized in a two-branch structural framework, where the appearance features are extracted in the appearance branch part by detecting each frame, followed by a feature extraction model, which utilizes a feature set to store the features of the last 100 frames of each trajectory set.

With the new detection, the minimum cosine distance between the feature set $R_{i}$ of the *i*th trajectory and the feature $f_{i}$ of the *j*th detection is calculated as shown in Equation (4), with $f_{j}$ denoting the feature vector of the *j*th detection and $f_{k}^{\left(i\right)}$ denoting the feature vector of the corresponding trace, as the feature vector that retains the past *k* successful traces.

(4) $$d\left(i,j\right)=\min\left\{1-f_{j}^{T}f_{k}^{\left(i\right)}|f_{k}^{\left(i\right)}\in R_{i}\right\},$$

This distance is used as the matching cost in the association process. In the motion branch, the Kalman filtering algorithm is used to predict the position of the trajectory in the current frame. The Marxian distance is then used to measure the spatio-temporal difference between the trajectory and the detection. Deepsort uses this motion distance to filter out unlikely associations. However, the Kalman filter used by Deepsort is not robust and ignores information on the detection noise scale.

It then undergoes a matching cascade algorithm that treats the association task as a series of subproblems, rather than a global assignment problem. The core idea is to provide greater matching priority to the more familiar objects. Each association subproblem is solved using the Hungarian algorithm. However, as the tracker becomes more powerful, the matching cascade algorithm becomes more robust to confusable associations; thus, the additional prior constraints will limit the accuracy of matching.

In order to solve the problems of the ordinary Kalman filter becoming easily disturbed by the quality detection and that it ignores the information on the detection noise scale, this paper replaces the ordinary Kalman filter with the NSA Kalman filter [74] algorithm and introduces an adaptive calculation of the noise covariance ${\tilde{R}}_{k}$, as shown in Equation (5).

(5) $${\tilde{R}}_{k}=\left(1-c_{k}\right)R_{k},$$

where $R_{k}$ is a predetermined constant measurement noise covariance, $c_{k}$ is the detection confidence score under state *k*, and instead of using only the appearance feature distance, both appearance and motion information are considered in the matching process.

The cost matrix *C* is the weighted sum of the appearance cost $A_{a}$ and the action cost $A_{m}$, as in Equation (6).

(6) $$C=\lambda A_{a}+\left(1-\lambda \right)A_{m},$$

where the weight factor λ is set to 0.98. In addition, to solve the problem that additional prior constraints will limit the matching accuracy, the ordinary global linear assignment is used instead of the matching cascade.

The algorithm improvement also includes updating the appearance state $e_{i}^{t}$ of the *i*th trajectory at frame *t* in an exponential moving average (EMA) fashion, as shown in Equation (7).

(7) $$e_{i}^{t}=\alpha e_{i}^{t-1}+\left(1-\alpha \right)f_{i}^{t},$$

where $f_{i}^{t}$ is the appearance embedding of the current matching detection and α=0.9 is the momentum term. The EMA update strategy not only improves the matching quality, but also reduces the time loss.

**Figure 4:**
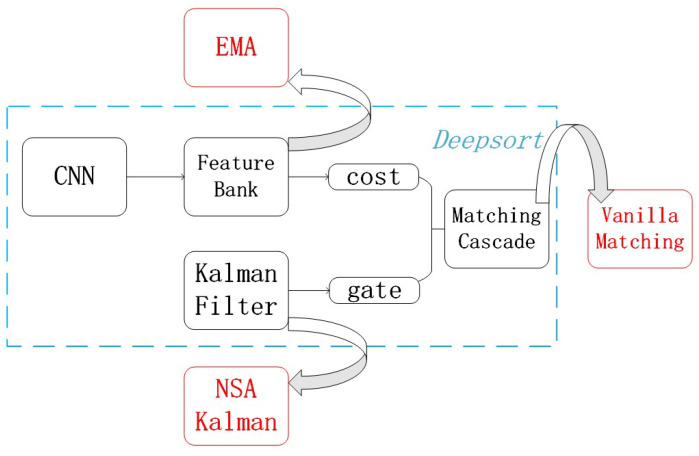
Deepsort Improvement Strategy Map.

In summary, all the improved strategies are shown in Figure 4 compared with the pre-improvement situation.

### 4.3. Pedestrian Re-Identification

The Fastreid algorithm uses a feature extraction model with a .pth suffix, which is the model file of Pytorch. This introduces the ONNX (Open Neural Network Exchange) deep learning model format, which optimizes the network inference speed and makes the model network structure more fine-grained.

The model files are converted from those with a .pth suffix to model files with an .onnx suffix according to the model conversion program.

The YOLOv5-Lite, Deepsort, and Fastreid algorithms are integrated into the pedestrian detection and re-identification system. Through the visual analysis and reasoning of the real-time video, it is found that the detection and reasoning speed of the experimental video is very slow, and the frame jamming phenomenon is more serious when each frame of the video is detected. Considering that the system may be bloated, some codes need to be optimized to improve the detection frame rate.

The pedestrian detection is changed to inter-frame detection, i.e., all pedestrians in the video are detected by the YOLOv5-Lite detection model in every other frame, and the frame rate module is added to the real-time video visualization interface to display the frame rate, which provides convenience for the model to make comparison tests based on the frame rate in the process of continuous iterative improvement.

## 5. Experiment Results

All comparison tests were conducted in the following hardware and software environments.

- Operating system: Linux
- Python version: 3.8.10
- Number of CPU: 24
- Number of GPU: 1
- GPU type: NVIDIA GeForce RTX 3060

The pedestrian detection dataset used for YOLOv5-Lite model training consists of a total of 3900 images containing only labeled pedestrians (19,000 pedestrians in total), of which 550 are used as the test set and 550 as the validation set; all data are stored in the YOLO format before training, with a training batch_size of 16, with each being trained for 150 sessions. In addition to this, to eliminate the bias in the dataset, we also conducted a comparative experiment on the performance of our model on an additional dataset, which we will describe in detail in the next experiments.

Experiments on multi-target tracking were conducted on datasets MOT15, MOT16, and MOT17Det, which are a series of datasets from the Multi-Target Tracking MOT Challenge series that provides a measure of multi-target detection tracking methods.

### 5.1. YOLOv5-Lite Experimental Analysis

The evaluation indicators of the comparison test for target detection are detailed below.

For recall, which assesses the ability of the model to find all positive samples, the higher the indicator, the better the model, and the formula is (8). TP means the true positive rate, which is the number of instances which are relevant and which the model correctly identified as relevant and FN means the false negative rate, that is the number of instances which are relevant and which the model incorrectly identified as not relevant.

(8) $$Recall=\frac{TP}{TP+FN}$$

Precision, which is the proportion of correctly predicted samples out of all predicted samples in the model, the higher the indicator, the better the model, as shown in Equation (9). FP means the false positive rate, which is is the number of instances which are not relevant but which the model incorrectly identified as relevant.

(9) $$Precision=\frac{TP}{TP+FP}$$

The mean average precision (mAP), first calculated for each category and then averaged over them, is commonly used in target detection as mAP_0.5, the average mAP over thresholds greater than 0.5, and mAP_0.5:0.95, which denotes the average mAP over different IoU thresholds, starting from 0.5 to 0.95 at 0.05 intervals (0.5, 0.55, 0.6, 0.65, 0.7, 0.75, 0.8, 0.85, 0.9, 0.95) on the mean mAP, as shown in Equation (10).

(10) $$mAP=\frac{1}{\;N}\sum_{i}^{N}AP_{i}$$

The improved network models were trained on the above hardware and software platforms using the pedestrian detection dataset (a total of 3900 images containing only labeled pedestrians, including a total of 19,000 pedestrians); each was trained for 150 rounds, using mAP_0.5:0.95, precision, mAP_0.5, and recall as the model’s superiority and inferiority. The optimal model before and after the improvement is shown in Table 1, and the overall size of the improved model increased slightly with the input size of 640 × 640. The accuracy of the model increased by 3% from 0.89 to 0.92, indicating that the accuracy of the improved model has increased to a certain extent, and compared with other algorithms, there is a high level of improvement in the model size and accuracy, and the performance is better in the test set.

Subsequently, in order to verify the generalization ability and practical effect of the model, we performed comparison experiments on different pedestrian datasets for the improved YOLOv5-lite model before and after.

One of the datasets used in the comparison experiments is the WiderPerson dataset, which is a benchmark dataset for pedestrian detection in more crowded scenes; its images are selected from a variety of scenes and are no longer limited to traffic scenes. The other dataset is the CUHK Occlusion dataset, which is from the Chinese University of Hong Kong and contains pedestrian images from various scenes. The division of the two datasets is shown in the following Table 2.

Under the above dataset division, we trained YOLOv4, YOLOv3, the original YOLOv5-Lite model and the improved YOLOv5-Lite model with the same training parameters: batch_size = 16, epochs = 150. The trained precision, recall, and mAP_0.5, and mAP_0.5:0.95 results are shown in Table 3 and Table 4.

The experimental results show that the improved YOLOv5-Lite model is the best in terms of the precision metric on the dataset WiderPerson, reaching 77%. Additionally, on the dataset CUHK Occlusion, our model has the highest precision, reaching 93%, as well as the first in Recall value, reaching an excellent 80%, which is sufficient to prove that our model has a better detection capability and excellent performance for different datasets.

### 5.2. Deepsort Experimental Analysis

The evaluation metrics for the multi-objective tracking comparison test are as follows.

FP: The total number of false positives, the number of positive samples predicted by the model but false in reality. The number of FP in the whole video is the sum of the number of FP in each frame. The lower the indicator, the better the performance.

FN: The total number of false negatives, the number of negative samples predicted by the model. The number of FN in the whole video is the sum of the number of FN in each frame. The lower the index, the better the performance.

FM (Fragm): The total number of fragmentations; each tracking object is considered a fragmentation after the tracking is interrupted and resumed.

IDs: The total number of ID changes during tracking; the lower the indicator the better the performance.

GT (Ground Truth) refers to the real tag or real object.

MOTA: MOTA is used to evaluate the accuracy of tracking, negatively correlated with the number of occurrences of FNs, FPs, IDs, and Identity Switch (IDSW). Ground-truth tracks(gtDet) are represented as the sum of the gt of each frame in a video, using Equation (11), where the higher the performance of the indicator the better.

(11) $$MOTA=1-\frac{\left|FN|+|FP|+|IDSW\right|}{\left|gtDet\right|}$$

MOTP: This is used to measure the position error in the tracking process, where $C_{t}$ represents how many of the predicted tracks match the GT track successfully in frame *t*. Where $d_{t,i}$ stand for the distance measured by IOU or the Euclidean distance between the trajectory *i* and GT. If the IOU is greater than a certain threshold or the Euclidean distance is less than a certain threshold, it is regarded as matching. MOTP pays more attention to the detection quality than evaluating the tracking effect. The higher the index, the better the performance. The formula is as follows:

(12) $$MOTP=\frac{\sum_{t,i}d_{t,i}}{\sum_{t}c_{t}}$$

MT (Mostly Tracked trajectories): The number of GT trajectories with more than 80% of the total frames successfully tracked, where the higher the indicator, the better the performance.

ML (Mostly Lost trajectories): The number of GT trajectories where the number of successfully tracked frames is less than 20% of the total number of frames, and the lower the indicator, the better the performance.

IDP: The fraction of correctly identified detections, where the higher the indicator is the better, as shown in Equation (13). Identity true positives (IDTPs) correspond to the overlapping parts of trajectories. Identity false negatives (IDFNs) represent the area that does not intersect with the matching trajectories and remaining unmatched trajectories. Unmatched trajectories are referred to as Identity False Positives (IDFPs).

(13) $$IDP=\frac{IDTP}{IDTP+IDFP}$$

IDR: Recall value for correct identification, where the higher the performance of the indicator the better, as shown in Equation (14).

(14) $$IDR=\frac{IDTP}{IDTP+IDFN}$$

IDF1: The reconciled mean value of IDP and IDR, where the higher the performance of the indicator the better, as in Equation (15).

(15) $$IDF1=\frac{2}{\frac{1}{IDP}+\frac{1}{IDR}}=\frac{2IDTP}{2IDTP+IDFP+IDFN}$$

MODA (Multiple Object Detection Accuracy) and MODP (Multiple Object Detection Precision) are also applied in target detection to measure the performance of the model, where *t* denotes the frame, $m_{t}$ denotes the number of missed detects in frame *t* (missed detects), and $fp_{t}$ denotes the number of false positives (false alarms). c(m) represents the custom loss function for missed detections, and $c_{f}$ represents the custom loss function for false alarms. $N_{G}^{t}$ represents the number of ground truth objects in frame *t*, as shown in Equation (16).

(16) $$MODA\left(t\right)=1-\frac{c_{m}\left(m_{t}\right)+c_{f}\left(fp_{t}\right)}{N_{G}^{t}}$$

(17) $$OverlappingRatio=\sum_{i=1}^{N_{mapped}^{t}}\frac{\left|G_{i}^{\left(t\right)}\cap D_{i}^{\left(t\right)}\right|}{\left|G_{i}^{\left(t\right)}\cup D_{i}^{\left(t\right)}\right|}$$

In OverlappingRatio, $G_{i}^{\left(t\right)}$ represents the ground truth label of the *i*-th object in frame *t*. $D_{i}^{\left(t\right)}$ represents the result of the detection. $N_{mapped}^{t}$ represents the total number of objects detected in this frame, as shown in Equation (17). Dividing the overlapping ratio by the quantity $N_{mapped}^{t}$ provides the indicator MODP, as shown in Equation (18).

(18) $$MODP\left(t\right)=\frac{OverlapRatio}{N_{mapped}^{t}}$$

FAR (False Acceptance Rate): The number of false recognitions per frame, where the lower the index, the better the performance.

RCll (Recall): Recall rate, as shown in Equation (8).

PRCn (Precision): Accuracy rate, as shown in Equation (9).

We conducted comparative experiments on the MOT15, MOT16 and MOT17 datasets, respectively, and the results are shown in Table 5, Table 6, Table 7, Table 8 and Table 9. Regardless of the dataset, our model achieves the highest performance in the metrics of IDF1, IDP, and IDR, indicating the best detection score for correct identification, in addition to our model’s MOTA and MOTP, which all show the best results in the test. On dataset MOT16, our improved model achieves an impressive score of 99.8 in the MOTP metric, indicating that the precision of our multi-objective tracking is the highest in the current dataset compared to that of other models of the experiment. In addition, on the MOT15 dataset, our IDs metric was reduced to 134, indicating that our improved model greatly reduces the occurrence of the ID-switch phenomenon; at the same time, our improved model also has a substantial reduction in its FP and FN metrics, the false alarm rate of the model has been improved, and the performance and accuracy reached the best results at this stage.

The analysis shows that after the algorithm improvement, the tracking performance and accuracy of Deepsort improved substantially, and the performance is better on the same data set than after the improvement.

### 5.3. Fastreid Experimental Analysis

The model with the .pth suffix before the conversion and the model with the .onnx suffix after the conversion were used to extract features from 12 pedestrian photos, and the time used for calculation was 2.76 s before the improvement, while the feature extraction time of the improved model with the onnx format was only 0.67 s, which represents a significant reduction in feature extraction time.

In the dataset VERI-Wild compared the Fastreid model with other popular pedestrian re-identification models. As can be seen in Table 10, the mAP metric of the Fastreid model is consistently higher than that of other models, and its application to our system can achieve the desired accuracy results.

### 5.4. System Experimental Analysis

After the improvement of the system, pedestrian detection is changed to inter-frame detection. Under the experimental hardware and software platform of 4.3.1, the detection frame rate of the experimental video is increased from 7∼10 frames to 15∼25 frames, and the detection speed is significantly improved, and the real-time effect can be achieved.

### 5.5. System Showcase

**Figure 5:**
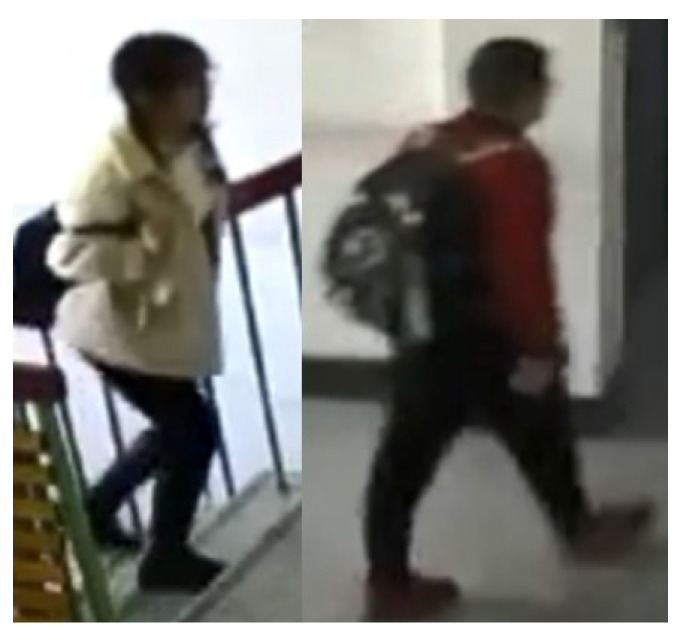
The images of pedestrians to be detected, from left to right, are named bag, red.

**Figure 6:**
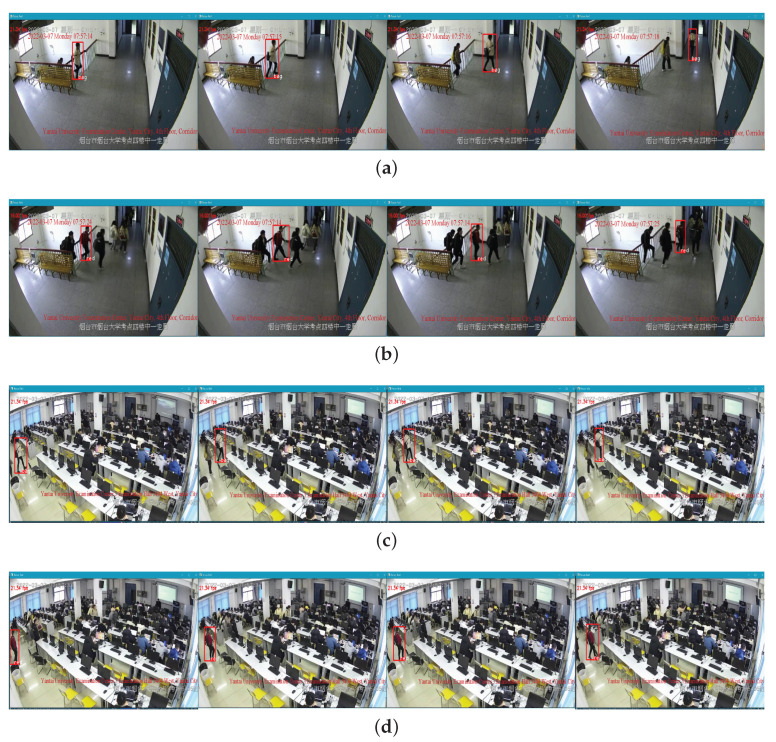
Real-time pedestrian re-identification in different area cross camera. (**a**,**b**): Display of pedestrian re-identification in area 1. (**c**,**d**): Display of pedestrian re-identification in area 2.

Figure 5 shows two images of pedestrians to be searched for. Under the experimental hardware and software platform of 4.3.1, the effects of real-time video detection, tracking and re-identification are shown in Figure 6, and the average frame rate is 20∼25 FPS. The whole process from the appearance to the disappearance of the target is correctly tracked, as seen in Figure 6a. Moreover, it is still accurately re-identified and tracked in the new scene in Figure 6c. In Figure 6b, the person wearing red clothes can be tracked perfectly even if the level of confusion increases. When this person appears in Figure 6d, he is first accurately re-identified, and then the whole walk is tracked. Therefore, the proposed system can achieve a real-time accurate search and localization of specific pedestrians across time, regions and cameras.

## 6. Conclusions

In this paper, we introduce an efficient and powerful framework for real-time video pedestrian detection, re-identification and tracking. Using the improved YOLOv5-Lite algorithm with higher accuracy, the improvement as well as mainstream recognition algorithms, and the BiFPN module to solve the possible inconsistency of multi-scale feature information of the model, the detection performance of the improved YOLOv5-Lite algorithm is validated against current academic benchmarks on our adopted dataset, showing that the improved algorithm is even better. Using the improved Deepsort tracking algorithm, we introduce the NSA Kalman filtering algorithm to eliminate the interference of quality detection, the EMA update strategy to improve the matching quality to reduce the time loss, and use the ordinary global linear assignment instead of the matching cascade to solve the problem that additional prior constraints can limit the matching accuracy, and validate our improved effect using the MOT16 dataset. The results show that our method has a considerable performance improvement in each tracking metric compared to that before the improvement, and our method is more advanced and robust. Finally, we also made corresponding improvements to the Fastreid algorithm and the overall system, which were well tested and showed that our improvements were quite effective.

## Figures and Tables

**Table 1:** Comparison of detection results with other methods.

Model	Input Size	Flops	Size	Precision	Recall	mAP_0.5	mAP_0.5:0.95	FPS
YOLOv5	640×640	16.5 G	96.1	0.89	0.91	0.95	0.83	28
tph-YOLOv5	640×640	16.2 G	92.7	0.87	0.90	0.96	0.84	25
YOLOv5-Lite	640×640	15.6 G	1.10	0.89	0.93	0.97	0.87	42
Ours	640×640	15.7 G	1.11	0.92	0.93	0.97	0.87	41

**Table 2:** Experimental data division for different data sets.

Dataset	Total Number	Train Set	Test Set
WiderPerson	13,382	9000	4382
CUHK Occlusion	1063	850	213

**Table 3:** Comparison of the detection results with other methods on the dataset WiderPerson.

Model	Input Size	Flops	Precision	Recall	mAP_0.5	mAP_0.5:0.95
YOLOv4-tiny	640×640	6.48 G	0.27	0.61	0.52	0.24
YOLOv3-tiny	640×640	13.0 G	0.75	0.54	0.62	0.32
YOLOv5-Lite	640×640	15.6 G	0.76	0.64	0.70	0.40
Ours	640×640	15.7 G	0.77	0.62	0.69	0.40

**Table 4:** Comparison of the detection results with other methods on the CUHK Occlusion dataset.

Model	Input Size	Flops	Precision	Recall	mAP_0.5	mAP_0.5:0.95
YOLOv4-tiny	640×640	6.48 G	0.28	0.52	0.42	0.20
YOLOv3-tiny	640×640	13.0 G	0.88	0.77	0.84	0.47
YOLOv5-Lite	640×640	15.6 G	0.92	0.76	0.88	0.52
Ours	640×640	15.7 G	0.93	0.80	0.88	0.52

**Table 5:** Comparison of tracking metrics on MOT15 dataset and deepsort algorithm.

Method	IDF1	IDP	IDR	RCll	PRCn	FAR	GT
Sort	45.9	55.4	39.2	56.0	79.2	1.07	500
Deepsort	41.1	43.5	39.0	54.5	60.8	2.55	500
Ours	86.6	87.1	86.1	97.5	98.7	0.09	500

**Table 6:** Comparison of tracking metrics on MOT15 dataset and deepsort algorithm (continued).

Method	MT	ML	FP	FN	IDs	FM	MOTA	MOTP
Sort	120	145	5871	17,573	544	984	39.9	72.8
Deepsort	114	195	14,023	18,150	500	1117	18.1	72.2
Ours	420	27	521	1007	134	82	95.8	99.8

**Table 7:** Comparison of tracking metrics on MOT16 dataset and deepsort algorithm.

Method	IDF1	IDP	IDR	RCll	PRCn	FAR	GT
Sort	29.7	45.0	22.2	37.6	76.3	2.43	517
Deepsort	37.4	74.7	24.9	31.3	94.0	0.42	517
Ours	93.6	93.9	93.3	98.9	99.6	0.09	517

**Table 8:** Comparison of tracking metrics on MOT16 dataset and deepsort algorithm (continued).

Method	MT	ML	FP	FN	IDs	FM	MOTA	MOTP
Sort	41	271	12,916	68,904	1090	1493	24.9	77.9
Deepsort	30	307	2214	75,817	239	1190	29.1	78.5
Ours	490	1	489	1170	258	45	98.3	99.8

**Table 9:** Comparison of tracking metrics on MOT17Det dataset and deepsort algorithm.

Method	RCll	PRCn	FAR	FP	FN	MODA	MODP
Sort	99.1	55.0	10.14	53,917	626	17.8	96.1
Deepsort	99.3	99.3	0.08	439	492	98.6	95.0
Ours	99.2	99.3	0.09	489	508	98.5	99.6

**Table 10:** Comparison of the state-of-the-art vehicle Re-Id methods on the VERI-Wild dataset.

Methods		Small		Medium		Large
	mAP	R-1	mAP	R-1	mAP	R-1
GoogLeNet [75]	24.3	57.3	24.2	53.2	21.5	44.6
DRDL [76]	22.5	57.0	19.3	51.9	14.8	44.6
FDA-Net [77]	35.1	64.0	29.8	57.8	22.8	49.4
MLSL [78]	46.3	86.0	42.4	83.0	36.6	77.5
Fastreid	87.7	96.4	83.5	95.1	77.3	92.5

## Data Availability

The datasets are available on Github at https://github.com/xuanwang-91/Framework-for-Pedestrian-Detection-Tracking-and-Re-identifcation.git accessed on 10 October 2022.
